# Mycotic Sphenopalatine Sinusitis With Concurrent Compression of the Optic Nerves and Chiasm and Severe Visual Impairment in A Horse

**DOI:** 10.1111/vop.70003

**Published:** 2025-03-07

**Authors:** Niklas Holz, José Suárez, Udo Hetzel, Antonella Rampazzo, Riccardo Stoppini

**Affiliations:** ^1^ Ophthalmology Section, Vetsuisse Faculty University of Zurich Zurich Switzerland; ^2^ Clinic for Diagnostic Imaging, Vetsuisse Faculty University of Zurich Zurich Switzerland; ^3^ Institute of Veterinary Pathology (IVPZ), Vetsuisse Faculty University of Zurich Zurich Switzerland

**Keywords:** equine, fungal, optic neuropathy, post‐retinal, sinusitis, visual impairment

## Abstract

A 15‐year‐old Swiss Warmblood gelding was presented to the ophthalmology service of Zurich University due to severe visual impairment. Ophthalmic and neurologic examinations were performed, raising suspicion of post‐retinal blindness. Standing contrast computed tomography (CT) of the head revealed a partially calcified, soft tissue attenuating mass in the sphenopalatine sinus with concurrent compression of the optic nerves and chiasm. Differential diagnosis included neoplasia and granulomatous disease. The horse was euthanized, and the head was subsequently examined by magnetic resonance imaging (MRI) followed by gross and histopathology. MRI showed compression of the optic nerves and chiasm. Histopathology revealed the formation of fibrous granulation tissue, osseous metaplasia, and pyogranulomatous inflammation in the sphenopalatine sinus. Periodic acid‐Schiff reaction and Grocott silver staining demonstrated branching septated filament hyphae and fungal spores. The optic nerves and chiasm were evident of mild neuronal atrophy, showing mild gliosis, vacuolation, and mild lympho‐plasmacytic inflammation. Mycotic sphenopalatine sinusitis should be considered as a more specific diagnosis for post‐retinal blindness in horses due to the compression of the optic nerve(s) and chiasm.

## 1 Introduction

Mycotic infections of the equine sinuses are infrequently reported and considered uncommon [1]. In a retrospective study with 200 horses, mycotic sinusitis accounted for only seven cases. All horses were characterized by purulent or mucopurulent discharge, and some showed facial swelling [2]. The pathogenesis is not fully understood, although hematogenous spread from guttural mycosis and inhalation of spores are proposed mechanisms.

Primary sinusitis, usually caused by bacteria, has been linked to optic nerve atrophy and blindness in horses [3]. Other reports of sinus disease with concurrent blindness include ossifying fibroma [4] and paranasal lymphoma [5]. Because the optic chiasm overlies the sphenopalatine sinus, this proximity may be responsible for the described pathological alterations. For a review of related anatomical structures, the reader is referred to McCann et al. describing the clinical anatomy of the equine sphenopalatine sinus in detail [6]. Sphenoid sinus mucoceles have been described as a rare occurrence in humans causing vision loss due to optic nerve compression. Expansion into adjacent tissues potentially leads to bone erosion of the optic canal. Surgical decompression and drainage have been performed, resulting in variable outcomes [7].

Guttural pouch mycosis can lead to profound blood loss due to erosion of the internal and external carotid or maxillary arteries, which may cause ischemia to the optic nerve. In the case series by Hardy et al. surgical management with arterial occlusion of the carotid and greater palatine arteries led to acute onset of blindness within 24–48 h. This was accompanied by a very poor prognosis to regain vision [8, 9]. Direct fungal spread via the cribriform plate secondary to mycotic osteomyelitis of the frontal bone has been described in a case report of a horse showing severe ataxia and absent menace responses [10]. Primary mycotic sinusitis was associated with infraorbital neuritis and neuralgia leading to trigeminal neuropathy and signs of headshaking in a case series of Fiske‐Jackson et al. Treatment led to resolution of neurological signs in 3/5 horses [11]. Pujol et al. published a case series of horses with suspected primary mycotic rhinitis and paranasal sinusitis [1]. The horses had clinical signs of mucopurulent nasal discharge, and some also had epistaxis. The conchofrontal and maxillary sinuses were most commonly affected (6/7 horses) with additional involvement of the sphenopalatine sinus in one case. *Aspergillus spp*. were isolated in 3/4 positive cultures. Surgical management via trephination, repeated debridement, and topical antifungals resulted in resolution of clinical signs in 5/6 horses [1]. Detailed descriptions of sinus disease cases and their associated complications will further improve our clinical understanding and offer more treatment options for mycotic infections of the equine sinuses [1, 11].

To the authors knowledge, this is the first documented case of mycotic sphenopalatine sinusitis involving the optic nerves and chiasm, leading to severe visual impairment in a horse. Therefore, our objective was to propose mycotic sinusitis as a more specific diagnosis for post‐retinal blindness in horses.

## 2 Case Report

A 15‐year‐old Swiss Warmblood gelding was referred to the ophthalmology department of Zurich University due to severe visual impairment. The owners reported disorientation and a severely reduced ability to navigate since being relocated to a darker stable 4 weeks prior. Three days prior to presentation, the horse was examined by an equine veterinarian who noted bilateral mydriasis and initiated topical therapy with Nepafenac 0.1% eye drops (Nevanac Susp Opht 0.1%, Alcon, Novartis Pharma Schweiz) and 2% Dorzolamide/0.5% Timolol eye drops (Cosopt Gtt Opht steril, Santen SA) both twice daily. The prescribed treatment was directed to a tentative diagnosis of uveitis with secondary glaucoma.

The horse was in a good body condition without a history of preexisting disease. Vital parameters were within normal limits. The head was symmetric, and no nasal or ocular discharge was noted. Percussion of the sinuses was within normal limits. However, this technique is not always reliable [12] and not all sinuses can be directly assessed. Involvement of the palatine and sphenoidal sinuses can only be indirectly determined with caudal maxillary involvement and fluid levels communicating with the bordering sinuses. A complete ophthalmic examination was performed, including slit‐lamp biomicroscopy (Kowa SL‐17; Kowa Company Ltd), rebound tonometry (TonoVet, Icare Finland Oy, Helsinki, Finland), indirect ophthalmoscopy (Omega 500; Heine) and fluorescein staining (Fluo strips, Contacare Ophthalmics and Diagnostics). Reflex testing demonstrated an absent menace response in the medial and lateral visual fields, while dazzle and pupillary light reflex (PLR) were still present in both eyes. Pupils were normal in size and shape. The intraocular pressure was 21 and 20 mmHg in the left and right eyes, respectively. The eyes were otherwise unremarkable. On neurologic examination, the patient's demeanor was calm and alert. Assessment of the cranial nerves was unremarkable, except for the absent menace response in both eyes. Outside in a light environment, the horse was able to navigate, but it oriented towards the person leading it and appeared moderately uncoordinated while moving over small obstacles. Generalized hypermetria was noted and interpreted as uncertainty at a walk because of the visual impairment, rather than being a true neurologic abnormality since the complete neurological examination was otherwise unremarkable. Moving the horse into dim light conditions of the examination room resulted in immediate disorientation and nervous behavior of the animal. The horse exhibited behavior consistent with vision loss.

**FIGURE 1:**
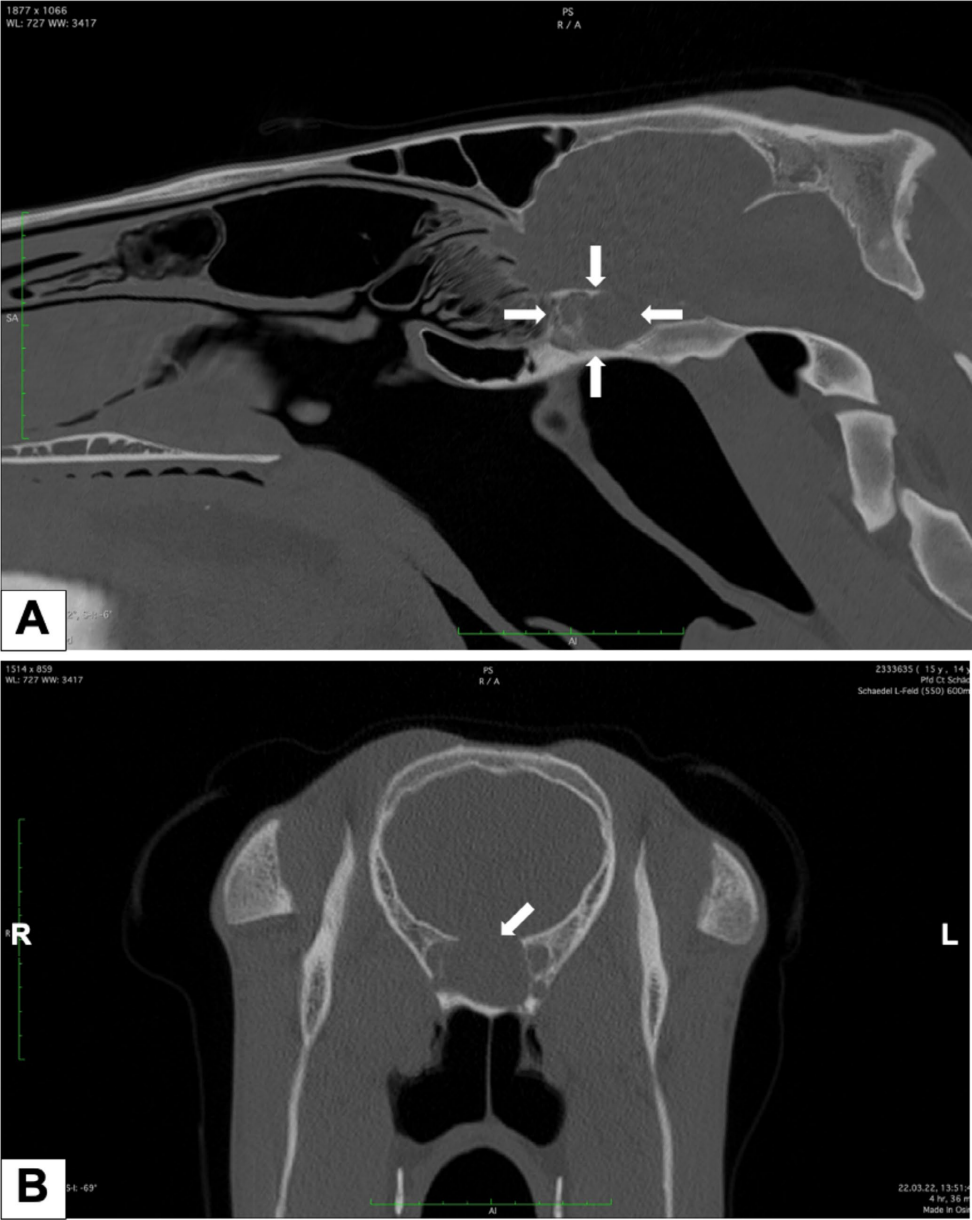
(A) sagittal head CT: A partially mineralized soft tissue‐attenuating mass in the sphenopalatine sinus; (B) transverse head CT: Bone atrophy of the presphenoid bone (bone algorithm).

CT imaging of the head with intravenous contrast medium (Omnipaque, 350 mg/mL Iohexol, GE Healthcare) was performed under standing sedation. The images (Figure 1) demonstrated complete obliteration of the sphenopalatine sinus lumen with a soft tissue attenuating space‐occupying lesion (30HU, 5 × 3 × 2.5 cm). The mass contained areas of mineral attenuation as well as a thin mineral‐attenuating capsule. Mild protrusion of the mass into the cranial vault was appreciated, displacing the hypophysis caudally and compressing the optic chiasm. Thinning of the adjacent presphenoid bone was observed, primarily affecting the laminae of the optic canal, orbital fissure, and alar canal. Typically well‐defined bony laminae dividing the sphenopalatine sinus were indistinguishable. The primary differential was neoplasia, although granulomatous disease could not be excluded. Treatment options would have needed a confirmed histologic diagnosis to be obtained via sinoscopic tissue sampling. Trephination of the conchofrontal bone would have allowed access to the lesion. The owners elected not to pursue further diagnostic steps and treatment, and the horse was euthanized. Postmortem examination of the head was performed with the owner's consent.

**FIGURE 2:**
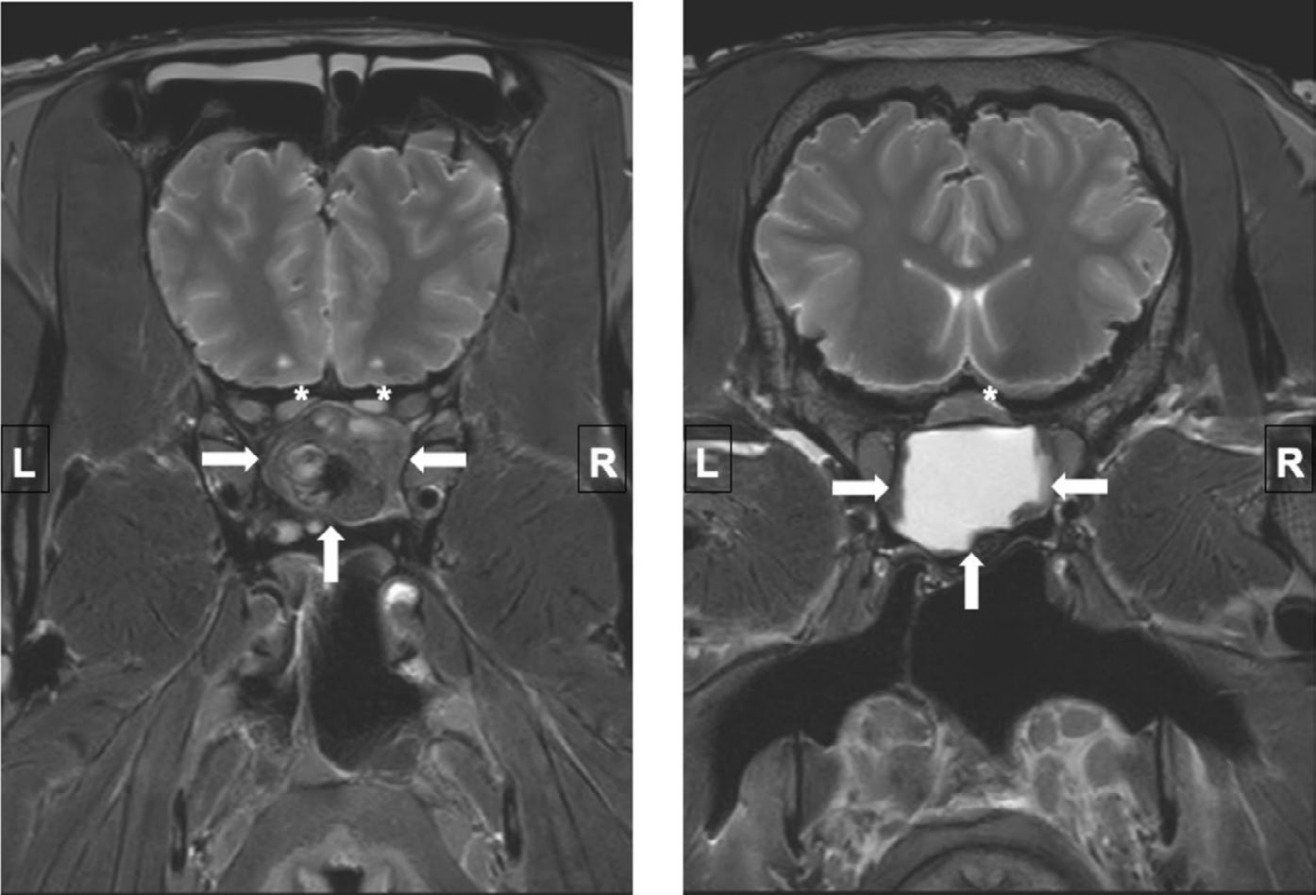
T2 TSE transverse MRI: Optic nerve compression rostral to (asterisks, left image) and at the level of the optic chiasm (asterisk, right image); space occupying lesion in the sphenopalatine sinus (arrows).

**FIGURE 3:**
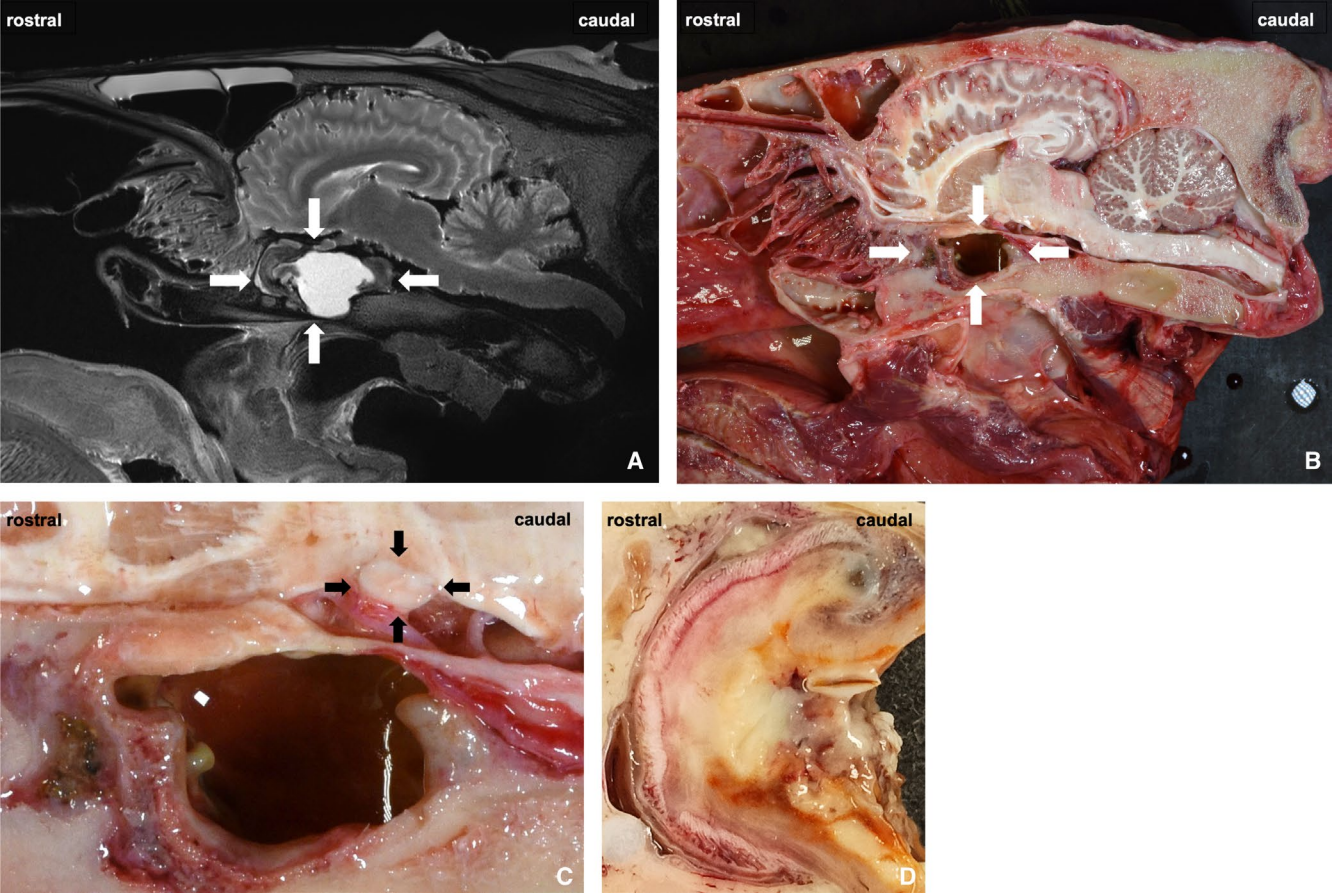
(A) T2 TSE sagittal MRI: Rostral part with heterogeneous T2 hypointense tissue, caudal part with homogeneous T2 hyperintense material representing fluid accumulation; (B) gross pathology: Mass lesion in situ (white arrows), rostral part with granulation tissue and peripheral ossification, caudal part with pus‐like surface coatings; (C) gross pathology: The optic nerve (black arrows) overlying the lesion appears flattened; (D) gross pathology: Rostral part of the lesion in detail.

MRI of the head was performed without contrast medium as part of the postmortem examination. The T2 TSE sagittal plane images (Figure 3A) showed a space‐occupying lesion composed of two different signal intensities. The rostral part of the mass consisted of heterogeneous T2 hypointense tissue, while the caudal part consisted of homogeneous T2 hyperintense material, suggestive of fluid. The T2 TSE transverse plane images (Figure 2) demonstrated signs of optic nerve compression with flattening of the optic nerves rostral to and at the level of the optic chiasm. The identified compression was directly overlying the space‐occupying lesion.

Gross pathology (Figure 3B,D) revealed a space‐occupying lesion in the sphenopalatine sinus with grossly distinct appearances rostrally and caudally. Rostrally, the lesion resembled granulation tissue with peripheral ossification, while caudally there was thickening of the mucosal lining with a purulent coating. The optic nerves overlying the lesion appeared flattened (Figure 3C). Macroscopically, there was no evidence of inflammatory changes in the eyes and the brain.

**FIGURE 4:**
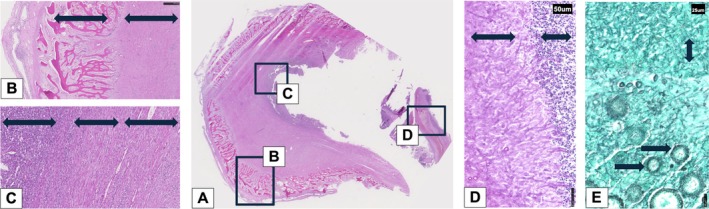
(A) section of the mass extending into the rostral part of the sinus; (B) fibrous granulation tissue (left arrow) and osseous metaplasia (right arrow); (C) chronic pyogranulomatous inflammation (left arrow), granulation tissue (middle arrow), fibrosis (right arrow); (D) PAS reaction with branching septated filamentous hyphae (upper arrow) and pyogranulomatous inflammation (lower arrow); (E) Grocott silver stain showing branching septated filamentous hyphae (double arrow) and fungal spores (single arrows).

**FIGURE 5:**
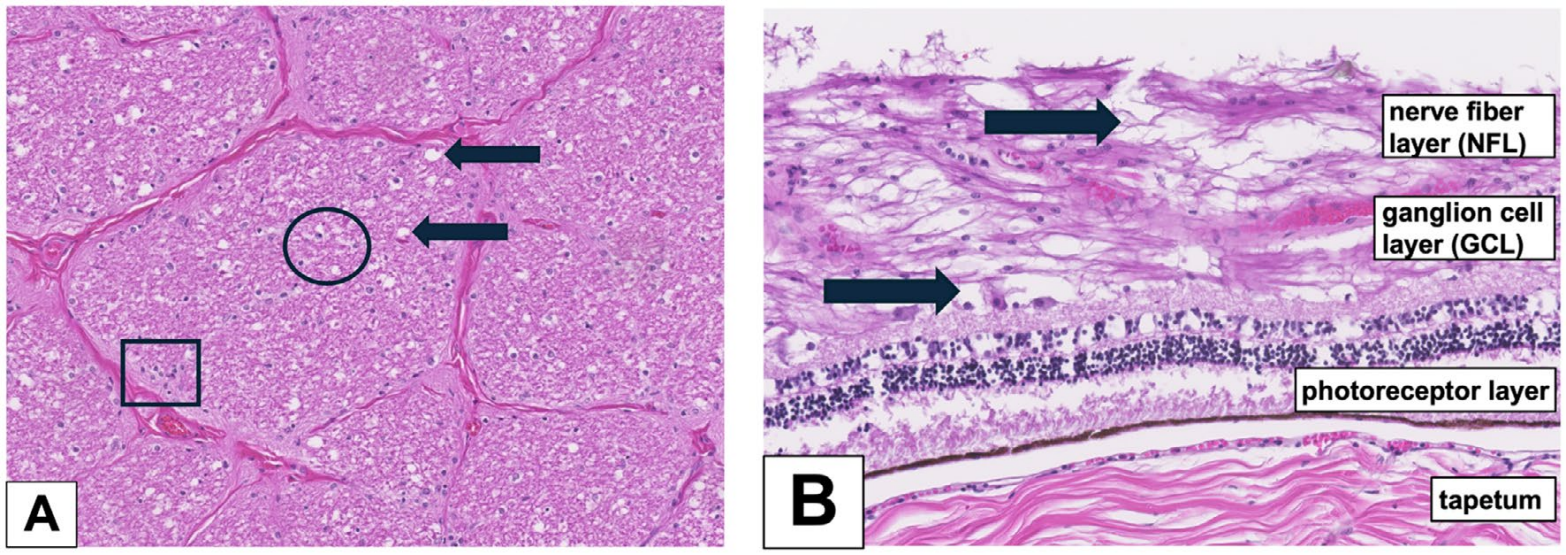
(A) optic nerve: Mild neuronal atrophy showing mild gliosis (circle), vacuolation (arrows), and mild lympho‐plasmacytic inflammation (rectangle); (B) retina: Mild retinal atrophy, NFL and GCL with vacuolation (arrows), and slight photoreceptor disorganization.

Histopathology was performed on relevant structures of the head, including the mass, globes, optic nerves, optic chiasm, cerebrum, cerebellum, brainstem, and medulla. Hematoxylin–eosin staining of the mass showed fibrous granulation tissue and osseous metaplasia demarcating an area with severe pyogranulomatous inflammation (Figure 4A–C). PAS reaction revealed the presence of branching, septate, filamentous hyphae. Grocott silver staining confirmed the presence of fungal hyphae and spores, which were morphologically consistent with *Aspergillus spp*. (Figure 4D,E). Sections of the optic nerves and optic chiasm were evidence of mild neuronal atrophy, showing mild gliosis, vacuolation, and mild lympho‐plasmacytic inflammation (Figure 5A). The retina of both eyes demonstrated signs of mild atrophy. The nerve fiber layer (NFL) and ganglion cell layer (GCL) showed vacuolar changes and slight photoreceptor disorganization, likely due to postmortem autolysis (Figure 5B). Sections of the globes and brain did not reveal evidence of inflammatory processes.

## 3 Discussion

The clinical signs of severe visual impairment are deemed consistent with a compressive chiasmatic and optic neuropathy concurrent with a chronic mycotic sphenopalatine sinusitis. Decreased to absent PLR would be expected in patients with a chiasmal lesion [13]. The referring veterinarian noted unresponsive bilateral mydriasis on initial examination, which was not appreciated 3 days later at our institution. Barnett et al. described a similar presentation of atypical visual impairment in their case series of three horses affected by sinusitis. One of the three horses described demonstrated absent reflexes on initial examination, although normal PLR was noted on re‐examination only 2 h later. Intermittent PLR function and blindness continued for 2 weeks until the owner elected for euthanasia in this case [3] Fluctuating levels of fluid accumulation seen with paranasal sinusitis may result in variable distension of the sphenopalatine sinus roof and subsequent optic nerve and chiasm compression, corresponding to inconsistencies in PLR and the ability to see. In our case, we reported the same inconsistencies.

Vision, menace response, dazzle, and PLR share common afferent pathways: the retina, the optic nerve, the optic chiasm, and the optic tract. Lesions within these structures may explain the visual behavior (reduced ability to navigate in daylight conditions and negative menace response) and the positive dazzle and PLR. The authors hypothesize that the mild neuronal atrophy with gliosis of the optic nerves and chiasm, and the lympho‐plasmacytic inflammation are responsible for the observed alterations. The damaged structures may have retained enough viable cells to elicit a dazzle and a PLR but not a menace response. The minimal degree of vision necessary to barely navigate is often lost after the loss of the menace response. The retinal function was not evaluated by electroretinogram (ERG) testing at the time of examination. Histologically, the retina demonstrated mild atrophy, which could be age‐related, while the observed vacuolation and photoreceptor changes are most likely related to post‐mortem autolytic changes. The authors hypothesize that at the time of our examination, the retinal function still allowed the horse to navigate in daylight but not in dim light. The atypical gait with hypermetria was associated with decreased to absent vision in our horse, which is supported by another study where a blindfolded mare demonstrated signs of hypermetria in all four limbs [3].

Histology of the optic nerves revealed mild neuronal atrophy showing signs of gliosis. Gliosis is characterized by hypertrophy of astrocytes replacing injured neurons. This is an unspecific response to tissue injury of the central nervous system and is believed to play a role in limiting edema by forming a glial scar. It may at the same time block regenerating axons from entering the damaged areas [14]. Gliosis is associated with different forms of optic atrophy, in our case a descending form originating at the level of the optic chiasm [15]. The chronic and severe sinus lesion in our case may have led to the signs of gliosis through compression, possibly resulting in ischemia and the release of proinflammatory cytokines to the optic nerve. While retinal changes (vacuolation and photoreceptor disorganization) were suspected to be postmortem autolytic changes, the optic nerves and chiasmatic gliosis are attributed to ante mortem structural changes. Since gliosis leads to glial scar formation with fibrosis, it seems unlikely that treatment would improve those structural changes. Ischemia secondary to optic nerve compression has been attributed to sphenoid sinus mucoceles leading to severe vision loss in humans [7].

Clinical presentation of nasal discharge and facial asymmetry in the equine patient typically raises suspicion for paranasal sinus disease [16]. In our patient, without typical signs of paranasal sinus disease and bilaterally absent menace response, the neuro‐ophthalmic lesion was localized at the level of the optic chiasm. The authors would like to point out that unless fluid levels are accumulating to the level of the maxillary sinus, nasal discharge is not necessarily expected with involvement of the sphenopalatine sinus only. Facial asymmetry is also less likely with primary sphenopalatine sinusitis and would typically be associated with disease processes affecting other sinuses. Therefore, care must be taken to avoid misleading diagnostic hypotheses in case of single sinus involvement.

Pre and postcontrast standing CT was the modality of choice to assess for intracranial lesions and proved to be a valuable diagnostic tool in this case. The use of CT imaging for horses affected by neurologic disorders has been validated in a retrospective study of 57 cases. Sogaro‐Robinson et al. showed that 4 out of 7 horses with an abnormal menace response or impaired vision had atypical findings on CT of the head [17]. Radiographically, the anatomical location of the sphenopalatine sinus causes unavoidable superimposition of bone, compromising its utility in evaluating the sinus [18]. This is supported by a retrospective study of Cissell et al. describing CT features of tumors in the nasal cavity and paranasal sinuses. By comparing CT features to radiographic findings, the authors concluded that radiographs were the least sensitive for detecting sphenopalatine masses. All tumors on CT were iso‐ or hypoattenuating except ossifying fibroma. While sinonasal neoplasia was always associated with bone lysis, only few horses with other sinonasal disease showed signs of bone destruction. In those other cases bone lysis was typically associated with a mass effect [19]. Tucker et al. concluded that CT is the only technique performed under standing sedation that is reliably able to assess the sphenoidal sinus [18]. In our case, boney margins between the sphenopalatine sinus and the base of the skull were hard to distinguish, presenting a limitation of the chosen imaging modality. Partial volume averaging results from an off‐centered dense object protruding partly into the *x*‐ray beam resulting in a shading artifact [20]. Blurring of the object margins may account for the artifact of bone loss on CT and especially thin bony plates may appear lytic. While any tissue mass has the potential to cause pressure atrophy of surrounding bone structures, neoplasia was the most frequent finding affecting the sphenopalatine sinus in middle aged to older horses [18].

Neoplastic lesions of the nasal cavity and paranasal sinuses may demonstrate a variable degree of mineralization depending on the specific type of tumor and have been reported for osteoma, osteosarcoma, odontoma, cementoma, nasal adenocarcinoma, and ossifying fibroma [19, 21]. While sinonasal neoplasia is typically associated with detectable signs of bone lysis, space‐occupying lesions with non‐neoplastic etiology such as sinus cysts or ethmoid hematomas have been described to cause bone destruction through pressure atrophy [19]. Mycotic lesions by *Aspergillus spp*. and *Penicillium spp*. have been associated with focal or diffuse sinusitis and bone erosion [12]. In our case, the mycotic lesion was of soft tissue and mineral‐attenuating density on CT. This is comparable to a recent case report describing an ossifying fibroma [4]. To aid the clinical decision‐making process, biopsy and histopathology would have been imperative to confirm the suspected etiology. Treatment rationale and prognosis should be based on an accurate diagnosis obtained by combined CT and sinoscopic tissue sampling [1, 11, 16, 18]. Veterinary reports are scarce, but treatment of mycotic rhinitis and paranasal sinusitis in horses without neurologic signs has been described [1, 12].

Another limitation of CT as an imaging modality is the reduced sensitivity of detecting intra‐axial changes, as shown by Lacombe et al. In this retrospective study, six out of seven cases with a normal CT showed abnormal histopathological findings of the brain [22]. MRI may overcome this limitation, but standing MRI of the equine head has not been described yet. The risk of general anesthesia in horses is up to 20 times higher than for small animals, and mortality rates of up to 1.1% have been reported [23]. Disorientation due to loss of vision was proposed as one reason for a significantly higher risk of unsatisfactory recovery after ophthalmic surgery [24]. Catastrophic limb fracture following a diagnostic imaging procedure was the reason for euthanasia in one horse in a study by Curto et al. [23]. Therefore, the risk of general anesthesia must be outweighed by the information gained [25]. Increased costs of a general anesthesia compared with a standing sedation also present an economic factor to consider. As such, MRI was not the first choice of diagnostic modality in the presented clinical case.

Dr. Freeman described trephination with mechanical debridement under endoscopic guidance in combination with lavages and topical antimycotics [12]. A case series of Pujol et al. reported complete resolution of mucopurulent nasal discharge in horses with suspected primary mycotic rhinitis and paranasal sinusitis [1]. Treatment consisted of repeated surgical debridement and topical application of an antifungal agent. Regression of mycotic plaques observed via sinoscopy and complete resolution of clinical signs were observed within 1–5 months in 5/6 horses. The authors concluded that the disease can be treated effectively with a good long‐term prognosis [1]. The results are encouraging but cannot be directly extrapolated to our case. Also, in the case series of Pujol et al. the sphenopalatine sinus was only affected in 1/7 cases, and the infection mainly involved the conchofrontal and maxillary sinus. Another study with five horses suffering from mycotic sinusitis with suspected trigeminal neuropathy showed successful resolution of clinical signs of headshaking in three out of five horses. The proposed pathogenesis was infraorbital neuritis and neuralgia caused by the fungal infection. According to this study, treatment of fungal sinus disease is challenging, and resolution of clinical signs was not consistent with resolution of the mycotic infection. The same study concluded that erosion of the bone overlying the infraorbital nerve makes successful treatment more difficult to achieve [11]. Owners and treating veterinarians should be aware that despite effective treatment, recurrence of a mycotic infection is possible, and euthanasia should be considered in cases where tissue destruction becomes extensive [12]. Sphenoid sinus mucoceles are a rare occurrence in humans leading to compressive optic neuropathy. Expansion with consequent mass effect on surrounding tissues, including bone erosion of the optic canal in the sphenoid sinus, has been described. Visual impairment can be as severe as absent light perception. Treatment through rapid surgical decompression has been performed in humans with sinus mucoceles. Visual outcomes varied from irreversible vision loss to complete recovery. Interestingly, time to surgical intervention did not correlate with a better prognosis of vision [7].

The authors would like to discuss one limitation of this case report. During fundic examination, there was no evidence of retinal abnormalities, raising suspicion of post‐retinal pathology. To confirm retinal function, an ERG may have been performed but was not done due to the high suspicion of post‐retinal disease. The available information for the retina of this horse includes the ophthalmoscopically normal fundic examination and the histological findings. Histologically, the retina showed signs of mild atrophy and vacuolic changes of the NFL and GCL. Variations of the retinal architecture and the ratio of ganglion cells have been described in normal eyes of older horses, which may contribute to the seen histologic changes [26]. The slight photoreceptor disorganization in the presented case is likely due to autolytic changes, since the MRI was performed postmortem, which delayed fixation of the globes. Large globes will take longer to cool after death, and once being put in formalin, the relatively thick sclera slows penetration of the fixative. This increases their vulnerability to autolytic changes. Intravitreal injection of formalin may help with preserving the anatomy, but still, results are variable. In mice, the outer nuclear layer becomes less compact, and the internuclear space increases. Delayed fixation by 4–16 h after death resulted in nuclear pyknosis and expansion of the ganglion cell layer with vacuolated spaces. The described time frame and the histologic findings match our case. Still, extrapolation of these results has to be done with caution since globe sizes are very different in rodents compared with horses [27].

The specific fungal species in the present study could not be identified. Mycotic culture from swabs taken during gross histology examination yielded negative results. Panfungal PCR has been successfully performed from mycotic plaque material in another case series, but results of a panfungal PCR examination on paraffined tissue samples in this study yielded negative results [11]. Morphologically, the fungal hyphae in this case are most consistent with Aspergillus *spp*., which has been described as the most commonly isolated genus of fungi in equine sinus disease by other authors [1, 2, 10].

## 4 Conclusion

Mycotic sphenopalatine sinusitis should be considered as a differential for post‐retinal blindness in horses. The severe visual impairment in our case is likely due to the permanent compressive damage to the optic nerve and chiasm, while the inconsistent episodes of PLR deficits may be due to transitory sinus distension and subsequent variable compression of the optic nerves and chiasm. Combined soft tissue and mineral attenuation of the lesion on CT may closely resemble the recently described features of an ossifying fibroma. Advanced diagnostic imaging with CT or MRI and sinoscopic tissue sampling for histology may allow for an accurate diagnosis and guide treatment rationale.

## Author Contributions

**Niklas Holz:** investigation, methodology, project administration, visualization, writing – original draft, writing – review and editing. **José Suárez:** investigation, methodology, writing – review and editing. **Udo Hetzel:** investigation, methodology, writing – review and editing. **Antonella Rampazzo:** methodology, writing – review and editing. **Riccardo Stoppini:** investigation, methodology, supervision, writing – review and editing.

## Ethics Statement

Animal owners provided written informed consent for the described procedure(s) and publication of data.

## Conflicts of Interest

The authors declare no conflicts of interest.

## Data Availability

Data sharing not applicable to this article as no datasets were generated or analysed during the current study.
